# Accuracy of spinal orthopaedic tests: a systematic review

**DOI:** 10.1186/1746-1340-14-26

**Published:** 2006-10-31

**Authors:** Rob Simpson, Hugh Gemmell

**Affiliations:** 1 Anglo-European College of Chiropractic, 13-15 Parkwood Road, Bournemouth, UK

## Abstract

**Background:**

The purpose of this systematic review was to critically appraise the literature on the accuracy of orthopaedic tests for the spine.

**Methods:**

Multiple orthopaedic texts were reviewed to produce a comprehensive list of spine orthopaedic test names and synonyms. A search was conducted in MEDLINE, MANTIS, CINAHL, AMED and the Cochrane Library for relevant articles from inception up to December 2005. The studies were evaluated using the tool for quality assessment for diagnostic accuracy studies (QUADAS).

**Results:**

Twenty-one papers met the inclusion criteria. The QUADAS scores ranged from 4 to 12 of a possible 14. Twenty-nine percent of the studies achieved a score of 10 or more. The papers covered a wide range of tests for spine conditions.

**Conclusion:**

There was a lack of quantity and quality of orthopaedic tests for the spine found in the literature. There is a lack of high quality research regarding the accuracy of spinal orthopaedic tests. Due to this lack of evidence it is suggested that over-reliance on single orthopaedic tests is not appropriate.

## Background

An orthopaedic test is defined as a procedure designed to place functional stress on isolated tissue structures thought to be responsible for the patient's pain or dysfunction [1]. All orthopaedic tests achieve this either by stretching, compressing or contracting (commonly at the same time) certain tissue structures. Generally, stretching manoeuvres elicit dysfunction in ligaments, capsules and nerves; contractive forces assess muscles and tendons; compressive manoeuvres assess cartilage, bone and nerves [2].

Determining a diagnosis or differential diagnoses is dependent upon the examiner's awareness of clinical signs and symptoms, physical examination, knowledge of possible pathology, mechanisms of injury, palpatory skills and ability to perform provocative tests correctly [3,4]. The clinical usefulness of a provocative orthopaedic test is largely determined by the accuracy with which it identifies its target dysfunction [5]. Therefore, information on the accuracy of orthopaedic tests, signs or manoeuvres would be beneficial, as the clinician could then select the most accurate test(s) out of the possible hundreds available.

The ideal orthopaedic test would always give a positive result in those with the disorder tested for (true-positive), and a negative result in those without the condition being tested for (true-negative). It is, therefore, necessary to consider sensitivity and specificity of the tests. Sensitivity is the proportion of those with the target disorder in whom the test result is positive. Specificity is the proportion of those without the target disorder in whom the test result is negative [5].

The purpose of this study was to determine the accuracy of spinal orthopaedic tests through a systematic review of the methodological quality of papers.

## Methods

### Search methods

A search was conducted in MEDLINE, MANTIS, CINAHL, AMED and the Cochrane Library for relevant articles from inception up to December 2005 using the following strategy [6]:

1. sensitivity OR specificity OR screening OR "false positive" OR "false negative" OR accuracy OR "predictive value" OR "predictive values" OR "reference standard" OR roc OR likelihood

2. spine OR vertebrae OR thoracic OR lumbar OR cervical OR sacroiliac

3. diagnostic test OR orthopaedic OR orthopedic OR test OR physical exam

Multiple orthopaedic texts were reviewed in order to produce a comprehensive list of orthopaedic test names and synonyms. We also delineated a list of diagnoses related to the spine. We then refined the search by using specific spine diagnoses and specific orthopaedic test names with: #1 AND #2 AND #3.

These citations were then retrieved and reviewed using the inclusion/exclusion criteria. In addition, the references cited in the papers were then hand-searched for appropriate studies. Each primary author from all the studies was used in another search using MEDLINE to make sure any other appropriate papers were not missed.

### Selection

Only studies in the English language were included. Papers were selected if they reported sensitivity and specificity values, and results were reported as single test results and not combined values based on multidimensional tests with reporting a single value for the multidimensional approach as a whole. The diagnostic procedure had to be described in sufficient detail for its replication. The test had to be a physical examination procedure and not a method of special imaging. These tests had to be orthopaedic procedures and not tests for determining spinal manipulable lesions. The tests also had to be conducted on humans.

Two reviewers read all abstracts, independently of each other. Full text articles were retrieved that could not be excluded based on title and abstract. These articles were read and checked for inclusion by the two reviewers independently. Where disagreements occurred, these were resolved through consensus.

### Quality assessment

The included articles were assessed for their quality by using the "Quality assessment for diagnostic accuracy studies" (QUADAS) tool [7]. The quality items are shown in Table 1. Two reviewers independently assessed each study for quality of methodology and where disagreement occurred, the assessment was discussed and consensus reached.

**Table 1:** The QUADAS tool questions for methodological assessment of diagnostic studies. [6]

1. Was the spectrum of patients representative of the patients who will receive the test in practice?
2. Were selection criteria clearly described?
3. Is the reference standard likely to correctly classify the target condition?
4. Is the time period between reference standard and index test short enough to be reasonably sure that the target condition did not change between the two tests?
5. Did the whole sample or a random selection of the sample, receive verification using a reference standard of diagnosis?
6. Did patients receive the same reference standard regardless of the index test result?
7. Was the reference standard independent of the index test (i.e. the index test did not form part of the reference standard)?
8. Was the execution of the index test described in sufficient detail to permit replication of the test?
9. Was the execution of the reference standard described in sufficient detail to permit its replication?
10. Were the index test results interpreted without knowledge of the results of the reference standard?
11. Were the reference standard results interpreted without knowledge of the results of the index test?
12. Were the same clinical data available when test results were interpreted as would be available when the test is used in practice?
13. Were uninterpretable/intermediate test results reported?
14. Were withdrawals from the study explained?
Each item is scored as yes, no or unclear.

### Data extraction

Study characteristics of the included articles were extracted. To gain an understanding of the accuracy of the orthopaedic test, we focused on the sensitivity and specificity of the test in question.

Where there was more than one study available for the specific orthopaedic test the mean of the test for sensitivity and specificity was calculated.

## Results

Our initial search of the online databases yielded 362,058 references. Using the refined search terms resulted in 144 references. Reviewing the reference lists resulted in 6 additional abstracts. In total 150 articles were retrieved in full text and 21 articles were included in this review. The main reason for exclusion was lack of reporting of sensitivity and specificity.

The papers covered a wide range of tests designed to detect conditions ranging from sacroiliac joint pain and posterior pelvic pain since pregnancy to meningitis and disc herniation. Eight papers evaluated orthopaedic tests of the cervical spine, two for the thoracic spine and 11 relating to the lumbopelvic region.

The scores for the methodological quality of the studies [8-28] ranged from 4 to 12 out of a possible 14 points (Table 2). None of the papers achieved the highest score of 14; however, 29% scored 10 or more.

**Table 2:** QUADAS scores for spinal orthopaedic tests

**Author**	**Condition**	**QUADAS Score**
Laslett et al [8]	Sacroiliac Joint Pain/Dysfunction	12
Laslett et al [9]	Sacroiliac Joint Pain/Dysfunction	11
Shah & Rajshekhar [10]	Soft Cervical Disc Prolapse	11
Wainner et al [16]	Cervical Radiculopathy	11
Dreyfuss et al [13]	Sacroiliac Joint Pain/Dysfunction	10
Poiraudeau et al [14]	Lumbar Disc Herniation	10
Glaser et al [17]	Cervical Spinal Cord Compression	9
Mens et al [19]	Posterior Pelvic Pain since Pregnancy	9
Viikari-Juntura, Porras & Laasonen [20]	Cervical Radiculopathy	9
Cote et al [21]	Scoliosis	8
Cote et al [12]	Vertebrobasilar Blood Flow	8
Broadhurst & Bond [11]	Sacroiliac Joint Pain/Dysfunction	7
Siminoski et al [15]	Lumbar Vertebral Fractures	7
Kosteljanetz, Bang & Schmidt-Olsen [22]	Lumbar Disc Prolapse	7
Thomas et al [24]	Meningitis	7
Tong, Haig & Yamakawa [25]	Cervical Radiculopathy	7
Leboeuf [18]	Lumbopelvic Pain/Dysfunction	6
Lauder et al [23]	Lumbosacral Radiculopathy	6
Karachalios et al [26]	Scoliosis	5
Sandmark & Nisell [27]	Cervical Spine/Neck Pain	4
Albert, Godskesen & Westergaard [28]	Posterior Pelvic Pain since Pregnancy	4

Because of the heterogeneity of the tests, study populations, and reference standards, as well as the lack of studies for each area of the spine, statistical pooling was not possible.

### Sacroiliac studies

There were five studies that met the inclusion criteria for identifying sacroiliac joint pain (Table 3). We determined research for the accuracy of these tests to be mainly of high quality based on their QUADAS scores. Laslett et al. [8] suggested that due to the large size and lack of mobility of the sacroiliac joints, a large amount of force has to be exerted in the correct direction to adequately stress the structures. This is a potential source for false negatives. Also, if the stress is applied to the incorrect location, the SIJ may not be stressed and pain may arise from other tissues resulting in false positives. The clinician must also remember that the clinical examination may not be able to clearly diagnose a condition due to illness behaviours, severe pain, body size, structure and shape.

**Table 3:** Sensitivity and specificity of orthopaedic tests for SI pain/dysfunction

**Authors**	**No. of Subjects**	**Test**	**Sensitivity (%)**	**Specificity (%)**	**QUADAS Score**
Laslett et al [8]	48	SI Compression	91	83	
		SI Distraction	91	83	
		Thigh Thrust	91	83	12
		Gaenslen	91	83	
		Sacral Thrust	91	83	

Laslett et al [9]	48	SI Compression	69	69	
		SI Distraction	60	81	
		Thigh Thrust	88	69	11
		Gaenslen	53	71	
		Sacral Thrust	63	75	

Broadhurst & Bond [11]	40	Fabere	77*	50**	
		Posterior Shear	80*	69**	7
		Resisted Abduction	87*	65**	

Dreyfuss et al [13]	85	Gillet	43	68	
		Fabere	69	16	10
		Gaenslen	71	26	
		Thigh Thrust	36	50	

Leboeuf [18]	68	Fabere	10	86	
		SI Aggravation	20	59	
		Ely	44	83	6
		Yoeman	46	72	
		Sacral Base Spring	33	59	

* = based on at least 70% reduction in pain, ** = based on at least 90% reduction in pain

Broadhurst and Bond [11] similarly mentioned that the force needed to stress the SIJ is large and may strain surrounding tissues and joints such as the lumbar facet joints and the sacrospinous, interosseous and iliolumbar ligaments, resulting in false positives. However, both Laslett et al. [8] and Broadhurst and Bond [11] claimed that the commonly used tests for SIJ dysfunction do have diagnostic value, especially when used in the context of specific clinical reasoning. Conversely, Dreyfuss et al. [13] found tests that are commonly used to detect SIJ involvement to be of no diagnostic value on the basis of a 90% reduction in pain following an intra-articular block.

### Cervical radiculopathy studies

There were five studies that met the inclusion criteria for the identification of cervical radiculopathies (Table 4). We determined studies for the accuracy of Spurling's test and cervical distraction tests were mostly of high quality according to the QUADAS score. However, 2 studies of the Spurling's test were determined to be of low quality. Viikari-Juntura et al. [20] considered Spurling's test to be an important component in any examination of a patient with neck and arm pain due to its high specificity regardless of its low sensitivity. Tong et al. [25] agreed with Viikari-Juntura et al. [20] in that Spurling's test was not sensitive although specific, achieving 94% specificity in patients using the most stringent criteria for cervical radiculopathy. Spurling's test is therefore considered to be a good screening test to confirm a cervical radiculopathy, which is in agreement with the conclusions of Shah and Rajshekhar [10].

**Table 4:** Sensitivity and specificity of orthopaedic tests for cervical radiculopathy

**Authors**	**No. of Subjects**	**Test**	**Sensitivity (%)**	**Specificity (%)**	**QUADAS Score**
Shah & Rajshekhar [10]	50	Spurling	92	95	11

Wainner et al [16]	82	Spurling A	50	86	
		Spurling B	50	74	
		Shoulder Abduction	17	92	
		Valsalva	22	94	11
		Cervical Distraction	44	90	
		Median N. Tension	97	22	
		Radial N. Tension	72	33	

Viikari-Juntura et al [20]	43	Spurling	28 * 33 **	100	9
		Cervical Distraction	26		
		Shoulder Abduction	31 * 42 **		

Tong et al [25]	255	Spurling	30	93	7

Sandmark & Nisell [27]	75	Spurling	77	92	4
		Radial N. Tension	77	94	

* = applies to the right hand side, ** = applies to the left hand side

However, Sandmark and Nisell [27] found Spurling's test did not reproduce radicular pain, instead local pain in the musculoskeletal tissues occurred.

### Lumbar radiculopathy studies

There were three studies that met the inclusion criteria for identifying lumbar radiculopathies with orthopaedic tests (Table 5). We found research for the straight leg raise (SLR) test to be of moderate quality with regards to the QUADAS scores. Kosteljanetz et al. [22] emphasised the importance of interpreting a test result in the context of other tests results due to the interobserver variation with this test. Poiraudeau et al. [14] mentioned that the Bell and hyperextension test should be included in a systematic clinical assessment of patients with radicular pain due to these tests having better sensitivities than the crossed SLR and better specificities compared to the normal SLR tests.

**Table 5:** Sensitivity and specificity of orthopaedic tests for lumbar radiculopathy

**Authors**	**No. of Subjects**	**Test**	**Sensitivity (%)**	**Specificity (%)**	**QUADAS Score**
Poiraudeau et al [14]	78	Bell (E1)	37	63	
		Bell (E2)	49	62	
		Bell (E3)	53	63	
		Hyperextension (E1)	40	72	
		Hyperextension (E2)	46	59	
		Hyperextension (E3)	47	71	10
		SLR (E1)	77	39	
		SLR (E2)	83	36	
		SLR (E3)	79	37	
		Crossed SLR (E1)	31	89	
		Crossed SLR (E2)	32	74	
		Crossed SLR (E3)	35	86	

Kosteljanetz et al [22]	55	SLR	33	87	7
		Crossed SLR		100	

Lauder et al [24]	170	SLR	19	84	6

E1 = Examiner 1; E2 = Examiner 2; E3 = Examiner 3

### Posterior pelvic pain since pregnancy studies

There were only two studies found that met the inclusion criteria for identifying posterior pelvic pain since pregnancy (PPPP) (Table 6). Research for the active straight leg raise was of moderate quality, whereas research for the positive pelvic pain provocation, fabere, SIJ compression and gapping tests was of low quality based on the QUADAS scores. Albert et al. [28] recorded high sensitivities and specificities for the tests; however, it was the only paper found to meet the inclusion criteria for this systematic review evaluating those tests and it achieved a low QUADAS score of 4 out of 14.

**Table 6:** Sensitivity and specificity of orthopaedic tests for posterior pelvic pain since pregnancy

**Authors**	**No. of Subjects**	**Test**	**Sensitivity (%)**	**Specificity (%)**	**QUADAS Score**
Mens et al [19]	200	ASLR	87	94	9

Albert et al [28]	2269	Pelvic Pain Provocation	71	98	4
		Fabere	48	99	
		SI Compression	37	100	
		SI Gapping	18	100	

### Scoliosis studies

There were only two studies that met the inclusion criteria for the screening of scoliosis (Table 7). We found the research for the Adams forward bending test to be of moderate quality. Cote et al. [21] considered Adams test to be more sensitive that the scoliometer and is therefore considered the best non-invasive clinical test to evaluate scoliosis. Conversely, Karachalios et al. [26] concluded that the Adams test cannot be used as an effective tool for the early detection of scoliosis due to the high number of false positives.

**Table 7:** Sensitivity and specificity of orthopaedic tests for scoliosis

**Authors**	**No. of Subjects**	**Test**	**Sensitivity (%)**	**Specificity (%)**	**QUADAS Score**
Cote et al [21]	105	Adam's Forward Bend	92 *	60*	8
			73**	68**	

Karachalios et al [26]	2700	Adam's Forward Bend	87	93	5

* = Thoracic Curves, ** = Lumbar Curves

### Vertebrobasilar blood flow studies

There was only one study found which met the inclusion criteria with regards to using orthopaedic tests to detect potential vertebrobasilar arterial insufficiency (VBAI) (Table 8). We determined the one study for the extension-rotation test to be of moderate quality with a QUADAS score of 8. This study by cote et al.[12] found the test not to be a valid premanipulative clinical test for detecting reduced blood flow through the vertebral arteries and should therefore not be used for this purpose.

**Table 8:** Sensitivity and specificity of orthopaedic tests for VBAI

**Authors**	**No. of Subjects**	**Test**	**Sensitivity (%)**	**Specificity (%)**	**QUADAS Score**
Cote et al [11]	42	Extension-rotation (L)	0	67*	
			0	71**	8
		Extension-rotation (R)	0	86*	
			0	90**	

* = cut-off point 1, ** = cut-off point 2, (L) = Left hand side, (R) = Right hand side

### Meningitis studies

There was only one study found that met the inclusion criteria for the detection of meningitis using orthopaedic tests (Table 9). Our review found the limited research of the Kernig and Brudzinski tests to be of moderate quality. The only included study by Thomas et al. [24] found the sensitivity and specificity of Brudzinski's to increase with an increase in severity of meningitis, whereas the sensitivity of Kernig's decreased when it came to severe meningitis and the specificity remained about the same.

**Table 9:** Sensitivity and specificity of orthopaedic tests for meningitis

**Authors**	**No. of Subjects**	**Test**	**Sensitivity (%)**	**Specificity (%)**	**QUADAS Score**
Thomas et al [24]	297	Kernig	5*	95*	
			9**	96**	7
			0***	95***	
					
		Brudzinski	5*	95*	
			9**	96**	
			25**	96**	

* = suspected meningitis; ** = moderate meningitis; *** = severe meningitis

### Lumbar vertebral fracture studies

There was only one study that met the inclusion criteria for detecting lumbar vertebral fractures (Table 10). The only study we included in this review was for rib-pelvis distance which was of moderate quality. The study by Siminoski et al. [15] concluded that there was potential use of this test for the detection of lumbar vertebral fractures, although further research needs to be done.

**Table 10:** Sensitivity and specificity of orthopaedic tests for lumbar vertebral fracture

**Authors**	**No. of Subjects**	**Test**	**Sensitivity (%)**	**Specificity (%)**	**QUADAS Score**
Siminoski et al [15]	781	Rib-Pelvis Distance	19 (0)	98 (0)	
			46 (1)	88 (1)	7
			87 (2)	47 (2)	
			99 (3)	8 (3)	
			100 (4+)	0 (4+)	

Number in brackets indicates number of fingerbreadths.

### Cervical cord compression studies

There was only one study that met the inclusion criteria for detecting cervical spinal cord compression (Table 11). We found the limited research for the Hoffmann sign to be of moderate quality. The study by Glasser et al. [17] showed that, at present, this test is not an accurate screening tool for predicting the presence of cervical spinal cord compression of various aetiologies.

**Table 11:** Sensitivity and specificity of orthopaedic tests for cervical cord compression

**Authors**	**No. of Subjects**	**Test**	**Sensitivity (%)**	**Specificity (%)**	**QUADAS Score**
Glaser et al [17]	165	Hoffmann	58*	78*	
			33**	59**	9

* = results from spinal surgeon; ** = results from neuroradiologist

A summary of the QUADAS components for each of the studies is shown in Table 12.

**Table 12:** Individual QUADAS scores for included studies

Study	1. Spectrum	2. Selection	3. Ref Standard	4. Time Period	5. Verification	6. Same Ref Standard	7. Independent of Index Test	8. Index Test Execution	9. Ref Standard Description	10. Independent of Ref Standard	11. Ref Standard Independent of Index	12. Same Clinical Data	13. Uninterpretable Results	14. Withdrawals
Laslett [8]	Y	Y	Y	Y	Y	Y	Y	Y	Y	Y	Y	Y	N	N
Laslett [9]	Y	Y	Y	Y	Y	Y	Y	N	N	Y	Y	Y	N	Y
Shah [10]	Y	Y	Y	?	Y	Y	Y	Y	N	Y	Y	Y	N	Y
Broadhurst [11]	Y	Y	Y	?	N	N	Y	N	N	Y	Y	Y	N	N
Cote [12]	Y	Y	Y	?	Y	Y	Y	Y	N	N	N	Y	N	N
Dreyfuss [13]	Y	Y	Y	?	Y	Y	Y	Y	Y	?	?	Y	N	Y
Poiraudeau [14]	Y	Y	Y	?	N	N	N	N	?	Y	Y	Y	N	?
Siminoski [15]	Y	Y	Y	?	?	Y	Y	Y	?	?	Y	?	N	N
Wainner [16]	Y	Y	Y	Y	Y	Y	Y	Y	Y	Y	?	Y	N	N
Glaser [17]	Y	Y	Y	?	?	?	Y	Y	Y	Y	Y	Y	N	N
Leboeuf [18]	Y	Y	?	?	Y	?	?	Y	?	Y	Y	?	N	N
Mens [19]	Y	Y	Y	?	Y	Y	Y	Y	N	Y	?	Y	N	N
Viikari-Juntura [20]	Y	Y	Y	Y	N	Y	Y	Y	N	Y	Y	N	?	?
Cote [21]	Y	Y	Y	?	Y	Y	Y	Y	N	?	?	Y	N	?
Kosteljanetz [22]	Y	Y	Y	Y	N	?	Y	Y	N	Y	N	?	?	N
Lauder [23]	Y	N	Y	?	Y	?	Y	Y	N	?	?	Y	?	N
Thomas [24]	Y	Y	Y	?	Y	Y	Y	N	N	?	?	Y	?	N
Tong [25]	Y	N	Y	?	?	Y	Y	Y	N	Y	?	Y	?	?
Karachalios [26]	Y	Y	Y	?	N	N	Y	N	?	Y	N	?	N	N
Sandmark [27]	Y	Y	?	?	?	?	?	Y	?	N	Y	?	N	N
Albert [28]	Y	Y	?	?	?	?	?	Y	N	N	?	Y	?	N

Y = Yes; N = No; ? = Unclear

## Discussion

The purpose of this systematic review was to determine the quality of the research regarding accuracy of spinal orthopaedic tests. From the total number of initial papers collected, a minority met the inclusion criteria for this study. The 21 papers that were included showed a range of quality based on the QUADAS tool. A potential bias of the literature used relates to the fact that only papers published in English were used and that no unpublished papers were searched for.

An important result of this review is that there are few high quality studies in this area. All 21 papers used a spectrum of patients that were representative of the patients that would receive the test in practice. All but two papers [23,25] clearly described the selection criteria; however, there were no papers that did not have any mention of selection criteria. Nineteen of the papers used a reference standard that was currently considered to be the best method available to detect the target condition, whereas the remaining three papers [18,27,28] were considered unclear with regards to the reference standard used. Only five of the papers managed to rule out disease progression bias by clearly demonstrating that the time period between the reference standard and the index test was short enough to insure that there was no change in the status of the target condition [8,9,16,20,22]. The remaining sixteen papers were classified as unclear in this area.

Partial verification bias was avoided in 12 papers, in that the whole sample or a random selection of the sample received verification using a reference standard. There were four papers [11,20,22,26] in which partial verification bias was not avoided. The remaining five papers were unclear in this regard [15,17,25,27,28]. Differential verification bias was avoided in 16 papers, in that the patients received the same reference standard regardless of the index test result. One paper [11] did not avoid this and the remaining four papers [18,23,27,28] were unclear.

Eighteen papers avoided incorporation bias by having an index test that did not form part of the reference standard. The remaining three papers [18,27,28] were unclear in this area. Seventeen papers described the execution of the index test in sufficient detail to permit replication, whereas four papers did not. Conversely, three papers [8,13,17] described the execution of the reference standard in sufficient detail to permit its replication, whereas 14 papers did not and four papers were unclear [15,18,26,27].

Fifteen papers provided the same clinical data during interpretation of the test results as would be available when the test is used in practice. One paper [20] did not and five papers [15,18,22,26,27] were unclear. There were no papers that clearly reported any uninterpretable or intermediate results. Fifteen papers did not report these types of results whereas the remaining six papers [20,22-25,28] were unclear. Three papers [9,10,13] explained withdrawals from the study. Fourteen papers made no mention of withdrawals and the remaining four papers [14,20,21,25] were unclear in this regard.

## Conclusion

High quality research for the field of spinal orthopaedic tests, which are so commonly used in practice by many branches of manual medicine, is lacking. Due to this lack of research for any particularly excellent tests, one should continue to base clinical impressions on not just the result of a single test but multiple tests and a good history.

## Competing interests

The author(s) declare that they have no competing interests.

## Authors' contributions

HG conceived the research idea. HG and RS designed the study. RS and HG acquired the papers. HG and RS critically appraised the studies. RS and HG drafted the manuscript and have approved the final version for publication.
